# Diethyl 2,6-dimethyl­pyridine-3,5-dicarboxyl­ate at 100 K

**DOI:** 10.1107/S1600536809037349

**Published:** 2009-09-26

**Authors:** Wassima Ghalem, Ratiba Belhouas, Raouf Boulcina, Sofiane Bouacida, Abdelmadjid Debache

**Affiliations:** a Laboratoire des Produits Naturels d’Origine Végétale et de Synthèse Organique, PHYSYNOR, Université Mentouri-Constantine, 25000 Constantine, Algeria; b Faculté de Chimie, USTHB, BP32, El-Alia, Bab-Ezzouar, Alger, Algeria; c Unité de Recherche de Chimie de l’Environnement et Moléculaire Structurale, CHEMS, Université Mentouri-Constantine, 25000 Algeria.

## Abstract

In the structure of the title compound, C_13_H_17_NO_4_, the packing is stabilized by weak C—H⋯O and C—H⋯π inter­actions, resulting in the formation of a three-dimensional network.

## Related literature

For our studies on nitro­gen heterocycles, see: Debache *et al.* (2008*a* ▶,*b* ▶); Boulcina *et al.* (2007 ▶).
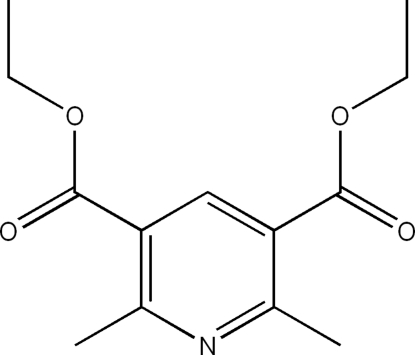


## Experimental

### 

#### Crystal data

C_13_H_17_NO_4_
*M* *_r_* = 251.28
Monoclinic, 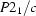

*a* = 4.5380 (6) Å
*b* = 15.440 (2) Å
*c* = 18.722 (2) Å
β = 90.502 (6)°
*V* = 1311.7 (3) Å^3^
*Z* = 4
Mo *K*α radiation
μ = 0.09 mm^−1^
*T* = 100 K
0.58 × 0.34 × 0.25 mm

#### Data collection

Bruker APEXII diffractometer
Absorption correction: multi-scan (*SADABS*; Sheldrick, 2002 ▶) *T*_min_ = 0.942, *T*_max_ = 0.977
9968 measured reflections
2977 independent reflections
2442 reflections with *I* > 2σ(*I*)
*R*_int_ = 0.038

#### Refinement

*R*[*F* ^2^ > 2σ(*F* ^2^)] = 0.048
*wR*(*F* ^2^) = 0.128
*S* = 1.04
2977 reflections
170 parameters
H atoms treated by a mixture of independent and constrained refinement
Δρ_max_ = 0.35 e Å^−3^
Δρ_min_ = −0.23 e Å^−3^

### 

Data collection: *APEX2* (Bruker, 2003 ▶); cell refinement: *SAINT* (Bruker, 2003 ▶); data reduction: *SAINT*; program(s) used to solve structure: *SIR2002* (Burla *et al*., 2003 ▶); program(s) used to refine structure: *SHELXL97* (Sheldrick, 2008 ▶); molecular graphics: *ORTEP-3 for Windows* (Farrugia, 1997 ▶) and *DIAMOND* (Brandenburg & Berndt, 2001 ▶); software used to prepare material for publication: *WinGX* (Farrugia, 1999 ▶).

## Supplementary Material

Crystal structure: contains datablocks global, I. DOI: 10.1107/S1600536809037349/hb5104sup1.cif

Structure factors: contains datablocks I. DOI: 10.1107/S1600536809037349/hb5104Isup2.hkl

Additional supplementary materials:  crystallographic information; 3D view; checkCIF report

## Figures and Tables

**Table 1:** Hydrogen-bond geometry (Å, °)

*D*—H⋯*A*	*D*—H	H⋯*A*	*D*⋯*A*	*D*—H⋯*A*
C9—H9*A*⋯O3^i^	0.97	2.51	3.2478 (18)	133
C6—H6*B*⋯*Cg*^ii^	0.96	2.67	3.4279 (16)	136

Symmetry codes: (i) 
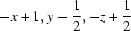
; (ii) 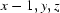. *Cg* is the centroid of the N1,C1–C5 ring.

**Fig. 1.**
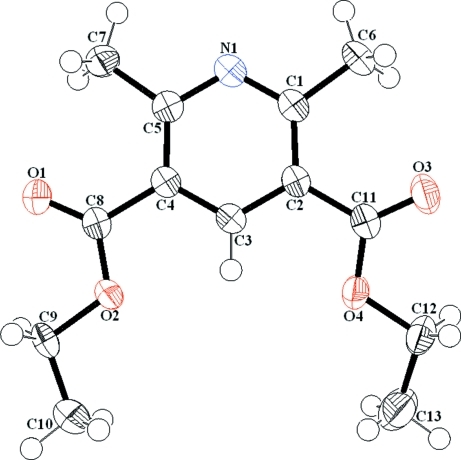
The molecular structure of (I): displacement ellipsoids are drawn at the 50% probability level.

**Fig. 2.**
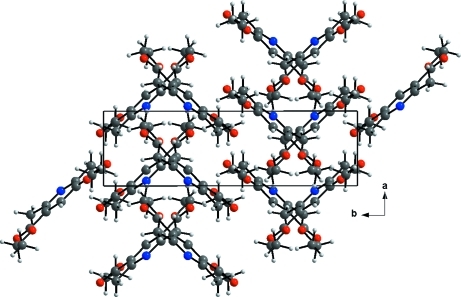
A diagram of the layered crystal packing of (I) viewed down the c axis.

**Fig. 3.**
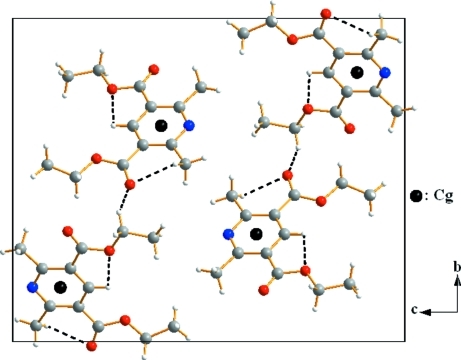
Unit cell of (I) showing hydrogen bond [C—H···O] as dashed line. Cg: is the controid of the nitrogen heterocyclic ring [N1—C5]

**Fig. 4.**
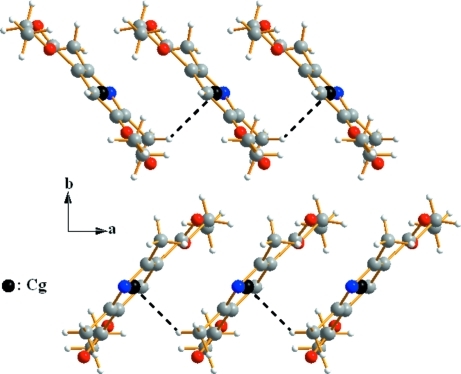
Part of crystal packing of (I) showing interactions between layers [C—H···π] as dashed line. Cg: is the controid of the nitrogen heterocyclic ring [N1—C5]

## supplementary crystallographic information

### Comment

In continuation of our interest in the synthesis and structure determination of nitrogen heterocyclic compounds (*e.g.* Boulcina *et al.*, 2007; Debache *et al.*, 2008*a*; Debache *et al.*, 2008*b*), herein, we report synthesis and crystallographic study of the title compound, (I), (Fig. 1), obtained from the oxidation of the corresponding 1,4-DHP.

The asymmetric unit of title compound contains a pyridine four times substituted by two dimethyl and two diethoxycarbonyl groups. As expected, The molecule is are approximately planar, the r.m.s. deviation for non-H atoms = 0.130Å with a maximum deviation from the mean plane = -0.3409 (19)Å for C13 atom.

The crystal structure can be described by two crossed layers which dihydropyridine ring is parallel to (-110) and (110) planes respectively (Fig.2).

The packing is stabilized by weak intermolecular interactions of C—H···O type (Figure 3) and the layers of dihydropyridine are linked together by C—H···π interactions (figure 4) involving the nitrogen heterocyclic ring (*Cg*), resulting in the formation of three dimensional network and reinforcing a cohesion of structure. Hydrogen-bonding parameters are listed in (table 1).

### Experimental

A 25-ml round-bottomed flask was charged with ethyl acetoacetate (2.0 mmol), 2-chloroquinoline-3-carboxaldehyde (1.0 mmol) and ammonium acetate (1.0 mmol), followed by 5 ml of water. The mixture was then refluxed until the reaction was completed (monitored by TLC). The reaction mixture was treated with brine solution, then extracted with ethyl acetate. After evaporation of the solvent, the crude yellow product was recrystallized from ethanol to give DHP in 85% yields. A solution of FeCl_3_.6H_2_O (0.1 mmol) in H_2_O (5 ml) was added to the obtained 1,4-dihydropyridines (1 mmol). The reaction mixture was stirred under refluxing until no starting material is detected. After the reaction was completed, the mixture was cooled to room temperature, quenched with 20 ml of H_2_O, neutralized with saturated aqueous solution of NaHCO_3_, and then extracted with ethyl acetate. The combined organic layer was dried over anhydrous sodium sulfate, and concentrated under a reduced pressure. Colourless blocks of (I) were obtained by a slow recrystallization from toluene.

### Refinement

In the final stages of refinement, all H atoms were localized in Fourier maps but introduced in calculated positions, with C—H distances of 0.96 and 0.97 for methylene and methyl H atoms, respectively, and refined using a riding model with *U*_iso_(H) values of 1.5*U*_eq_(C) for methyl H atoms and 1.2*U*_eq_(C) for methylene H atoms. Except for H3 atom were located in a difference Fourier map and refined isotropically. All non-H atoms were refined with anisotropic atomic displacement parameters.

### Crystal data

**Table:**

C_13_H_17_NO_4_	*F*(000) = 536
*M**_r_* = 251.28	*D*_x_ = 1.272 Mg m^−^^3^
Monoclinic, *P*2_1_/*c*	Mo *K*α radiation, λ = 0.71073 Å
Hall symbol: -P 2ybc	Cell parameters from 4103 reflections
*a* = 4.5380 (6) Å	θ = 2.5–27.4°
*b* = 15.440 (2) Å	µ = 0.09 mm^−^^1^
*c* = 18.722 (2) Å	*T* = 100 K
β = 90.502 (6)°	Block, white
*V* = 1311.7 (3) Å^3^	0.58 × 0.34 × 0.25 mm
*Z* = 4	

### Data collection

**Table:**

Bruker APEXII diffractometer	2977 independent reflections
Radiation source: fine-focus sealed tube	2442 reflections with *I* > 2σ(*I*)
graphite	*R*_int_ = 0.038
Detector resolution: 10.0 pixels mm^-1^	θ_max_ = 27.6°, θ_min_ = 2.5°
CCD rotation images, thin slices scans	*h* = −4→5
Absorption correction: multi-scan (*SADABS*; Sheldrick, 2002)	*k* = −19→19
*T*_min_ = 0.942, *T*_max_ = 0.977	*l* = −24→24
9968 measured reflections	

### Refinement

**Table:**

Refinement on *F*^2^	Primary atom site location: structure-invariant direct methods
Least-squares matrix: full	Secondary atom site location: difference Fourier map
*R*[*F*^2^ > 2σ(*F*^2^)] = 0.048	Hydrogen site location: difference Fourier map
*wR*(*F*^2^) = 0.128	H atoms treated by a mixture of independent and constrained refinement
*S* = 1.04	*w* = 1/[σ^2^(*F*_o_^2^) + (0.0665*P*)^2^ + 0.4357*P*] where *P* = (*F*_o_^2^ + 2*F*_c_^2^)/3
2977 reflections	(Δ/σ)_max_ < 0.001
170 parameters	Δρ_max_ = 0.35 e Å^−^^3^
0 restraints	Δρ_min_ = −0.23 e Å^−^^3^

### Special details

**Table:**

Geometry. All e.s.d.'s (except the e.s.d. in the dihedral angle between two l.s. planes) are estimated using the full covariance matrix. The cell e.s.d.'s are taken into account individually in the estimation of e.s.d.'s in distances, angles and torsion angles; correlations between e.s.d.'s in cell parameters are only used when they are defined by crystal symmetry. An approximate (isotropic) treatment of cell e.s.d.'s is used for estimating e.s.d.'s involving l.s. planes.
Refinement. Refinement of *F*^2^ against ALL reflections. The weighted *R*-factor *wR* and goodness of fit *S* are based on *F*^2^, conventional *R*-factors *R* are based on *F*, with *F* set to zero for negative *F*^2^. The threshold expression of *F*^2^ > σ(*F*^2^) is used only for calculating *R*-factors(gt) *etc*. and is not relevant to the choice of reflections for refinement. *R*-factors based on *F*^2^ are statistically about twice as large as those based on *F*, and *R*- factors based on ALL data will be even larger.

### Fractional atomic coordinates and isotropic or equivalent isotropic displacement parameters (Å^2^)

**Table:**

	*x*	*y*	*z*	*U*_iso_*/*U*_eq_	
C1	0.4395 (3)	0.39005 (9)	0.40522 (8)	0.0176 (3)	
C2	0.5062 (3)	0.39184 (8)	0.33193 (7)	0.0167 (3)	
C3	0.7120 (3)	0.33252 (8)	0.30658 (8)	0.0161 (3)	
H3	0.761 (5)	0.3320 (13)	0.2570 (12)	0.05*	
C4	0.8450 (3)	0.27347 (8)	0.35269 (7)	0.0163 (3)	
C5	0.7627 (3)	0.27417 (9)	0.42501 (7)	0.0179 (3)	
C6	0.2267 (3)	0.45056 (9)	0.44050 (8)	0.0224 (3)	
H6A	0.1927	0.4317	0.4886	0.034*	
H6B	0.0437	0.4506	0.4144	0.034*	
H6C	0.3074	0.508	0.4411	0.034*	
C7	0.8829 (4)	0.21296 (10)	0.48038 (8)	0.0248 (3)	
H7A	0.7855	0.2228	0.525	0.037*	
H7B	1.0907	0.2226	0.4864	0.037*	
H7C	0.8495	0.1544	0.4652	0.037*	
C8	1.0669 (3)	0.21071 (8)	0.32422 (7)	0.0166 (3)	
C9	1.3018 (3)	0.16026 (9)	0.21932 (8)	0.0206 (3)	
H9A	1.2478	0.1005	0.2286	0.025*	
H9B	1.4994	0.17	0.2378	0.025*	
C10	1.2891 (4)	0.17865 (12)	0.14082 (9)	0.0396 (5)	
H10A	1.0932	0.1679	0.1231	0.059*	
H10B	1.4255	0.1417	0.1165	0.059*	
H10C	1.3403	0.2381	0.1325	0.059*	
C11	0.3604 (3)	0.45328 (9)	0.28148 (8)	0.0185 (3)	
C12	0.2762 (4)	0.49029 (10)	0.16025 (8)	0.0268 (4)	
H12A	0.3523	0.5488	0.1643	0.032*	
H12B	0.0648	0.4918	0.1674	0.032*	
C13	0.3448 (6)	0.45354 (13)	0.08850 (10)	0.0491 (6)	
H13A	0.5544	0.4527	0.082	0.074*	
H13B	0.2549	0.4887	0.0521	0.074*	
H13C	0.2692	0.3956	0.0853	0.074*	
N1	0.5655 (3)	0.33183 (7)	0.44964 (6)	0.0189 (3)	
O1	1.2043 (2)	0.15896 (6)	0.35969 (6)	0.0236 (3)	
O2	1.0940 (2)	0.21896 (6)	0.25334 (5)	0.0201 (2)	
O3	0.2067 (2)	0.51374 (7)	0.29916 (6)	0.0286 (3)	
O4	0.4163 (2)	0.43422 (6)	0.21325 (5)	0.0238 (3)	

### Atomic displacement parameters (Å^2^)

**Table:**

	*U*^11^	*U*^22^	*U*^33^	*U*^12^	*U*^13^	*U*^23^
C1	0.0130 (6)	0.0164 (7)	0.0233 (7)	−0.0039 (5)	0.0008 (5)	−0.0038 (5)
C2	0.0142 (6)	0.0138 (6)	0.0219 (7)	−0.0025 (5)	0.0003 (5)	−0.0023 (5)
C3	0.0144 (6)	0.0148 (6)	0.0190 (7)	−0.0036 (5)	0.0008 (5)	−0.0025 (5)
C4	0.0136 (6)	0.0144 (6)	0.0208 (7)	−0.0039 (5)	0.0004 (5)	−0.0019 (5)
C5	0.0162 (7)	0.0161 (6)	0.0213 (7)	−0.0045 (5)	−0.0002 (5)	−0.0007 (5)
C6	0.0198 (7)	0.0206 (7)	0.0269 (8)	0.0005 (6)	0.0046 (6)	−0.0056 (6)
C7	0.0285 (8)	0.0245 (7)	0.0215 (8)	0.0017 (6)	0.0030 (6)	0.0034 (6)
C8	0.0152 (7)	0.0129 (6)	0.0218 (7)	−0.0030 (5)	−0.0001 (5)	−0.0018 (5)
C9	0.0178 (7)	0.0164 (6)	0.0276 (8)	0.0033 (5)	0.0031 (6)	−0.0047 (5)
C10	0.0495 (11)	0.0414 (10)	0.0280 (9)	0.0200 (9)	0.0102 (8)	−0.0003 (7)
C11	0.0143 (6)	0.0149 (6)	0.0264 (8)	−0.0032 (5)	0.0008 (5)	−0.0020 (5)
C12	0.0327 (9)	0.0193 (7)	0.0281 (8)	0.0036 (6)	−0.0081 (6)	0.0023 (6)
C13	0.0827 (16)	0.0362 (10)	0.0283 (10)	0.0183 (10)	−0.0110 (10)	0.0008 (8)
N1	0.0156 (6)	0.0184 (6)	0.0227 (6)	−0.0039 (5)	0.0021 (5)	−0.0022 (5)
O1	0.0253 (6)	0.0200 (5)	0.0255 (6)	0.0054 (4)	−0.0004 (4)	0.0016 (4)
O2	0.0209 (5)	0.0190 (5)	0.0205 (5)	0.0047 (4)	0.0036 (4)	−0.0014 (4)
O3	0.0287 (6)	0.0220 (5)	0.0351 (6)	0.0089 (5)	0.0045 (5)	−0.0002 (5)
O4	0.0301 (6)	0.0188 (5)	0.0225 (6)	0.0076 (4)	−0.0033 (4)	0.0004 (4)

### Geometric parameters (Å, °)

**Table:**

C1—N1	1.3483 (19)	C8—O2	1.3397 (17)
C1—C2	1.408 (2)	C9—O2	1.4584 (16)
C1—C6	1.5011 (19)	C9—C10	1.497 (2)
C2—C3	1.3945 (19)	C9—H9A	0.97
C2—C11	1.489 (2)	C9—H9B	0.97
C3—C4	1.3899 (19)	C10—H10A	0.96
C3—H3	0.96 (2)	C10—H10B	0.96
C4—C5	1.4079 (19)	C10—H10C	0.96
C4—C8	1.4986 (19)	C11—O3	1.2132 (17)
C5—N1	1.3466 (18)	C11—O4	1.3374 (18)
C5—C7	1.502 (2)	C12—O4	1.4586 (18)
C6—H6A	0.96	C12—C13	1.493 (2)
C6—H6B	0.96	C12—H12A	0.97
C6—H6C	0.96	C12—H12B	0.97
C7—H7A	0.96	C13—H13A	0.96
C7—H7B	0.96	C13—H13B	0.96
C7—H7C	0.96	C13—H13C	0.96
C8—O1	1.2089 (17)		
			
N1—C1—C2	121.38 (12)	O2—C9—C10	106.94 (12)
N1—C1—C6	114.49 (12)	O2—C9—H9A	110.3
C2—C1—C6	124.13 (13)	C10—C9—H9A	110.3
C3—C2—C1	117.97 (13)	O2—C9—H9B	110.3
C3—C2—C11	119.84 (12)	C10—C9—H9B	110.3
C1—C2—C11	122.18 (12)	H9A—C9—H9B	108.6
C4—C3—C2	120.54 (13)	C9—C10—H10A	109.5
C4—C3—H3	119.5 (13)	C9—C10—H10B	109.5
C2—C3—H3	119.9 (13)	H10A—C10—H10B	109.5
C3—C4—C5	118.34 (12)	C9—C10—H10C	109.5
C3—C4—C8	119.52 (12)	H10A—C10—H10C	109.5
C5—C4—C8	122.13 (12)	H10B—C10—H10C	109.5
N1—C5—C4	121.15 (13)	O3—C11—O4	122.98 (13)
N1—C5—C7	114.72 (12)	O3—C11—C2	124.78 (13)
C4—C5—C7	124.13 (13)	O4—C11—C2	112.24 (11)
C1—C6—H6A	109.5	O4—C12—C13	107.06 (13)
C1—C6—H6B	109.5	O4—C12—H12A	110.3
H6A—C6—H6B	109.5	C13—C12—H12A	110.3
C1—C6—H6C	109.5	O4—C12—H12B	110.3
H6A—C6—H6C	109.5	C13—C12—H12B	110.3
H6B—C6—H6C	109.5	H12A—C12—H12B	108.6
C5—C7—H7A	109.5	C12—C13—H13A	109.5
C5—C7—H7B	109.5	C12—C13—H13B	109.5
H7A—C7—H7B	109.5	H13A—C13—H13B	109.5
C5—C7—H7C	109.5	C12—C13—H13C	109.5
H7A—C7—H7C	109.5	H13A—C13—H13C	109.5
H7B—C7—H7C	109.5	H13B—C13—H13C	109.5
O1—C8—O2	123.74 (12)	C5—N1—C1	120.60 (12)
O1—C8—C4	125.24 (13)	C8—O2—C9	116.03 (11)
O2—C8—C4	111.02 (11)	C11—O4—C12	115.71 (11)
			
C1—C2—C3—C4	0.29 (19)	C9—C10—C11—C12	−176.91 (12)
C2—C3—C4—C5	1.11 (19)	C10—C11—C12—C13	13.4 (2)
C3—C4—C5—C6	−1.65 (14)	C11—C12—C13—N1	2.46 (9)
C4—C5—C6—C7	−179.72 (18)	C12—C13—N1—O1	−162.64 (16)
C5—C6—C7—C8	0.52 (11)	C13—N1—O1—O2	3.02 (4)
C6—C7—C8—C9	−172.90 (18)	N1—O1—O2—O3	3.67 (3)
C7—C8—C9—C10	167.30 (19)	O1—O2—O3—O4	−169.36 (7)
C8—C9—C10—C11	2.37 (14)		

### Hydrogen-bond geometry (Å, °)

**Table:**

*D*—H···*A*	*D*—H	H···*A*	*D*···*A*	*D*—H···*A*
C9—H9A···O3^i^	0.97	2.51	3.2478 (18)	133
C6—H6B···Cg^ii^	0.96	2.67	3.4279 (16)	136

Symmetry codes: (i) −*x*+1, *y*−1/2, −*z*+1/2; (ii) *x*−1, *y*, *z*.
